# Transcriptome and metabolome analysis in shoot and root of *Valeriana fauriei*

**DOI:** 10.1186/s12864-016-2616-3

**Published:** 2016-04-23

**Authors:** Yun Ji Park, Xiaohua Li, Seung Jae Noh, Jae Kwang Kim, Soon Sung Lim, Nam Il Park, Soonok Kim, Yeon Bok Kim, Young Ock Kim, Sang Won Lee, Mariadhas Valan Arasu, Naif Abdullah Al-Dhabi, Sang Un Park

**Affiliations:** Department of Crop Science, Chungnam National University, 99 Daehak-ro, Yuseong-gu, Daejeon, 305-764 Korea; Code Division, Insilicogen Inc., Suwon, Gyeonggi-do 441-813 Korea; Division of Life Sciences and Bio-Resource and Environmental Center, Incheon National University, Yeonsu-gu, Incheon, 406-772 Korea; Department of Food and Nutrition and Institute of Natural Medicine, Hallym University, Chuncheon, 200-702 Korea; Deptartment of Plant Science, Gangneung-Wonju National University, 7 Jukheon-gil, Gangneung-si, Gangwon-do 210-702 Korea; Biological and Genetic Resources Assessment Division, National Institute of Biological Resources, Incheon, 404-170 Korea; Department of Herbal Crop Research, National Institute of Horticultural and Herbal Science (NIHHS), Rural Development Administration (RDA), Bisanro 92, Eumseong, Chungbuk 369-873 Republic of Korea; Department of Botany and Microbiology, Addiriyah Chair for Environmental Studies, College of Science, King Saud University, P. O. Box 2455, Riyadh, 11451 Saudi Arabia

**Keywords:** *Valeriana fauriei*, Illumina HiSeq system, Digital gene expression, Terpenoid, Carotenoid, Phenylpropanoid

## Abstract

**Background:**

*Valeriana fauriei* is commonly used in the treatment of cardiovascular diseases in many countries. Several constituents with various pharmacological properties are present in the roots of *Valeriana* species. Although many researches on *V. fauriei* have been done since a long time, further studies in the discipline make a limit due to inadequate genomic information. Hence, Illumina HiSeq 2500 system was conducted to obtain the transcriptome data from shoot and root of *V. fauriei*.

**Results:**

A total of 97,595 unigenes were noticed from 346,771,454 raw reads after preprocessing and assembly. Of these, 47,760 unigens were annotated with Uniprot BLAST hits and mapped to COG, GO and KEGG pathway. Also, 70,013 and 88,827 transcripts were expressed in root and shoot of *V. fauriei*, respectively. Among the secondary metabolite biosynthesis, terpenoid backbone and phenylpropanoid biosynthesis were large groups, where transcripts was involved. To characterize the molecular basis of terpenoid, carotenoid, and phenylpropanoid biosynthesis, the levels of transcription were determined by qRT-PCR. Also, secondary metabolites content were measured using GC/MS and HPLC analysis for that gene expression correlated with its accumulation respectively between shoot and root of *V. fauriei*.

**Conclusions:**

We have identified the transcriptome using Illumina HiSeq system in shoot and root of *V. fauriei*. Also, we have demonstrated gene expressions associated with secondary metabolism such as terpenoid, carotenoid, and phenylpropanoid.

**Electronic supplementary material:**

The online version of this article (doi:10.1186/s12864-016-2616-3) contains supplementary material, which is available to authorized users.

## Background

**Fig. 1:**
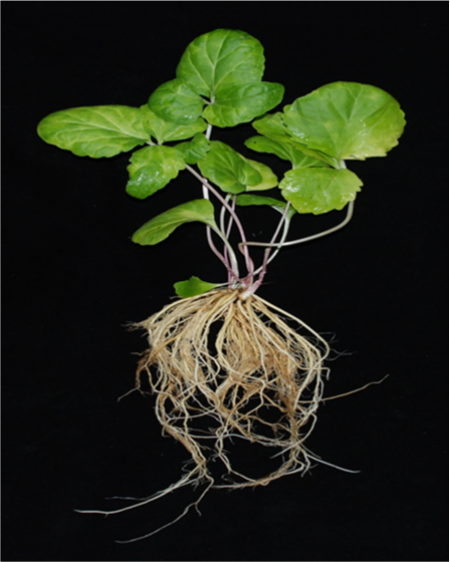
Photograph of 2-month-old seedling of *V. fauriei*

The genus *Valeriana*, containing over 250 species grown in Europe, Britain, and Asia [1, 2]. The roots of *Valeriana* plants are used mainly for medicinal purposes. The roots have been known to contain several photochemical constituents with pharmacological properties including hypnotic, sedative, antispasmodic, mild anodyne, hypotensive and carminative effects [3]. Among them, *Valeriana fauriei*, which is found widely in the northeast of China, South Korea, and Japan, has been used in folks for hundreds of years (Fig. 1) [4]. Researchers have isolated more than 150 compounds from *Valeriana* plants, such as monoterpenes, sesquiterpenes, iridoids, alkaloids, and so on [5]. It has been reported that flavonoids and alkaloids are primarily observed in the aerial parts and sesquiterpenes and iridoids noticed in rhizomes and roots [6, 7]. Among these constituents, valerenic acid is responsible for the sedative effects and its derivatives are produced in roots and rhizomes as principal compounds of *Valeriana* species [8, 9].

Terpenes, one of the major secondary metabolites in medicinal plants, have many volatile representatives such as isoprenes (C5), monoterpenes (C10), sesquiterpenes (C15), even some diterpenes (C20), and triterepenes (C30) [10]. Members of this family serve many important functions like protecting the plants from many insects, pest, herbivores and microbial pathogens such as bacteria and fungi [11]. All terpenes are assembled from the basic unit of isoprenes, isopentenyl diphosphate (IPP) and dimethylallyl diphosphate (DMAPP). These universal five carbon precursors are derived from two alternate biosynthetic pathways: mevalonate (MVA) pathway from acetyl-CoA and the 2-*C*-methylerythritol 4-phosphate (MEP) pathway from glycerol and pyruvate (Additional file 1) [12]. Recent report claimed that even though the operation of this pathway is independent, some metabolic connection were observed between the two pathways [13].

The MVA pathway initiates with the formation of acetoacetyl-CoA by acetocetyl-CoA thiolase (*AACT*). The HMG-CoA synthase (*HMGS*) catalyzes the synthesis of 3-hydroxy-3-methylglutaryl-CoA with one acetyl-CoA and one acetoacetyl-CoA. Next, HMG-CoA reductase (*HMGR*) synthesizes the MVA. Further, enzymes such as mevalonate kinase (*MK*), phosphomevalonate kinase (*PMK*), and mevalonate diphosphate decarboxylase (*MVD*) catalyze the formation of IPP. The first step in MEP pathway, pyruvate and glyceraldehyde-3-phosphate combined and produce 1-deoxy-D-xylulose 5-phosphate by DOXP synthase (*DXS*) which is mainly observed in plastids. Then, the conversion of 1-deoxy-D-xylulose-5-phosphate to 2-*C*-methyl-D-erythritol 4-phosphate is carried out by DOXP reductoisomerase (*DXR*). MEP is transformed into 1-hydroxy-2-methyl-2-(*E*)-butenyl 4-phosphate by the action of various catalytic enzymes. Enzyme (*E*)-4-hydroxy-3-methylbut 2-enyl diphosphate reductase (*HDR*) catalyzes the synthesis of IPP and DMAPP [12]. IPP and DMAPP are formed by the action of enzymes geranyl diphosphate synthase (*GPS*) and farnesyl diphosphate synthase (*FPS*), respectively [10]. IPP and DMAPP are the backbone for the synthesis of all terpenes.

Carotenoids, the natural C40 isoprenoid products, are essential hydrophobic plant compounds which contribute to yellow, orange or red color [14]. Carotenoids play an important role in photosynthesis, photomorphogenesis, and photoprotection. It is also mainly involved in the production of abscisic acid. The main carotenoid biosynthetic pathway was identified in many plants and studied extensively. The pathway begins with condensation of 3 molecules of geranyl-geranylpyrophosphate, a precursors from the upstream MEP pathway for production of 15-cis-phytoene catalyzed by phytoene synthase (*PSY*) (Additional file 2) [15]. Then, 15-cis-phytoene is converted to lycopene, the first yellow carotenoid, through desaturation reactions which are synthesized by both phytoene desaturase (*PDS*) and ζ-carotene desaturase (*ZDS*) [16]. Poly-cis lycopene to trans-lycopene is produced by the action of carotenoid isomerase (*CrtISO*) [17]. Next, carotenoid biosynthesis is branched to produce α- and β-carotene by enzymatic catalytic activity of two lycopene cyclases, lycopene β-cyclase (*LCYB*) and lycopene ε-cyclase (*LCYE*) [18]. α-carotene is hydroxylated into lutein by both β-ring hydroxylase (*CHXB*) and ε-ring hydroxylase (*CHXE*). In the other bransch, β-carotene is transformed into zeaxanthin; process is catalyzed by CHXB. Next, zeaxanthin epoxidase (*ZEP*) allows synthesis of violaxanthin from zeaxanthin. At last, the enzymatic activity of nine-cis-epoxycarotenoid dioxygenases (*NCEDs*) is responsible for the synthesis of abscisic acid as the final product [17]. Along the pathway, the oxidative activity of the specific enzymes generate apocarotenoids which is further degraded by carotenoid cleavage dioxygenases (*CCDs*) [19].

Phenolic compounds, widely distributed in higher plants, belong to one of the major classes of secondary metabolites including lignins, flavonols, isoflavonoids and anthocyanins [20]. These compounds contribute many important functional aspects of plant life such as UV sunscreens, pigments signaling. Additionally, accumulation of phenolic compounds is stimulated by biotic and abiotic responses. Currently, many researchers have been focused on the improvement of phenolic compounds in plants, because of its health promoting properties and curing properties to cancers, neurodegenerative diseases, cardiovascular diseases, osteoporosis and diabetes respectively [21]. Phenolic compounds are synthesized through the phenylpropanoid pathway and its biosynthesis starts with the condensation of the phenylalanine which is end product of shikimate pathway (Additional file 3). In the first step, deamination reaction involved by the action of phenylalanine ammonia-lyase (*PAL*) for the generation of *trans*-cinnamic acid [22]. Next, cinnamate 4-hydroxylase (*C4H*) and 4-coumaroyl CoA ligase (*4CL*) involved in the production of other intermediate metabolites [20]. In several side branches, *p*-coumarate acid 3-hydroxylase (*C3H*) converts *p*-coumaric acid to caffeic acid which is transformed to ferulic acid, carried out by the enzyme caffeate *O*-mehtyltransferase (*COMT*) [22]. Also, the enzyme hydroxycinnamoyl-CoA quinate hydroxyl cinnamoyl transferase (*HQT*) is involved in synthesis of *p*-coumaroylquinate from p-coumaroyl CoA which is then converted to chlorogenic acid by *C3H* [21]. Finally, chalcone synthase (*CHS*), catalyzes naringenin-chalcone, which is the derivative of the flavanoids. In downstream steps, various enzymes such as isomerases, reductases, and hydroxylases involved in the alternation of the basic flavonoid skeleton, leading to the different flavonoid subclasses [23].

Recently, whole transcriptome sequencing using next-generation sequencing (NGS) technologies, or RNA sequencing (RNA-Seq) has been widely used for the characterization of genes and their functions in secondary metabolite synthesis [24]. NGS technologies have efficiency due to its much higher levels of sensitivity, accuracy, dymamic-range of gene expression levels and fast run times (ranging from hours to days) compared to traditional low-throughput expressed sequence tag (EST) sequencing by Sanger technology [25, 26]. Also, many information such as quantitative gene expression, development of functional markets, quick insights into the specific gene space, comparative genomic studies and to isolate genes of interest [27, 28]. Among the common NGS platforms, including Illumina, Roche/454, SOLiD, and HelicosHeliScope, the Illumina HiSeq system has been commonly used because of its high throughput sequencing capacity which results in providing a higher coverage and low costs [29]. Despite these advantages, the sequence reads provide sufficient for *de novo* assemblies of full-length transcripts, except in the case of small classes of RNA [30]. For *Valeriana* family, only one transcriptome analysis from the *Valeriana officinalis* have been described in medicinal plant genomics resource (www.medicinalplantgenomics.msu.edu), whereas the transcriptome analysis of *V. fauriei* has not been investigated yet.

In this current study, we used the IlluminalHiSeq™ 2500 system to obtain the transcriptome in shoot and root of *V. fauriei*. We present data confirming that new sequencing technology can provide numerous insights into the molecular arrangement of secondary metabolite biosynthesis. In addition, we describe the identification of several full-length and partial-length cDNAs encoding genes related to terpenoid, carotenoid, and phenylpropanoid biosynthetic pathways. The transcripts levels of all genes were determined by real-time PCR and quantify the secondary metabolites with high-performance liquid chromatography (HPLC) to investigate the correlation between the transcriptional regulation of each biosynthetic gene and accumulation of each identified components. Till today no information has been provided the transcriptome characterization of *V. fauriei*.

## Results and discussion

### Sequencing and transcriptome assembly

**Fig. 2:**
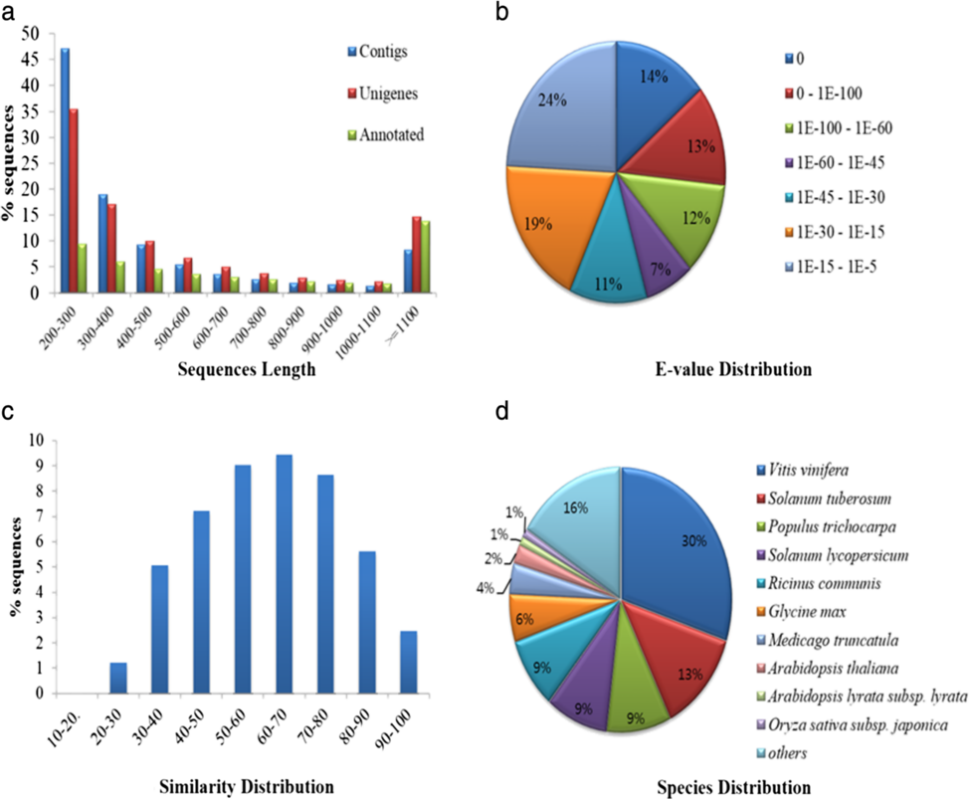
Summary for the assembly and annotations. **a** Sequence length distribution from contigs, unigenes, and annotations, **b** E-value distribution, **c** Similarity distribution, and (**d**) Species distribution. This figure shows the distributions of unigene BLASTX matches against UniProt database with E-value cut-off of 1e-5 and the proportions for each species

cDNAs prepared from root and shoot of *V. fauriei* were sequenced using Illumina Hiseq platform. As a result of sequencing, 346,771,454 raw reads were obtained from both samples (Table 1). Initially, total sequences were subjected to preprocessing, resulting in 291,047,351 (83.9 %) clean reads with 28,761,361,637 (82.1 %) total base. Preprocessed sequences were taken for *de novo* transcriptome assembly using CLC Assembly Cell software. In total, 22 different assemblies were generated and the each assembled contig set was qualified with the reference mapping as described in method section. Among those, assembly with word size 63 was selected as the best with respect to mapping result. In total, 23,797,128,049 (82.7 %) bases were mapped with coverage of 339.2 to the best assembled set with 143,401 contigs (70,145,151 bases). After redundancy removal by CAP3 assembly, 97,595 (61,543,817 bases) unique sequences were obtained ranging from 200 to 15,155 with an average length of 628.2 bases which were considered as a reference transcriptome for *V. fauriei* (Fig. 2a).

**Table 1:** Summary of the transcriptome assembly of *V. fauriei*

Sequences					
	Reads	Bases	AvgLen (bp)	MinLen (bp)	MaxLen (bp)
Raw Sequences	346,771,454 (100 %)	35,023,916,854 (100 %)	101	101.0	101
Processed Reads	291,047,352 (83.9 %)	28,761,361,637 (82.1 %)	98.7	90.0	101
Assembly					
Assembled Contigs	143,401 (100 %)	70,145,151 (100 %)	489.1	200.0	15,155
Unigenes	97,959 (68.3 %)	61,543,817 (87.7 %)	628.2	200.0	15,155

### Functional annotation

Final draft transcriptome was subjected to functional annotations by BLASTX mapping against to plant UniProt databases to obtain the biological terms with E-value cutoff 1e-5 (Table 2). Totally, 47,760 (48.7 %) sequences were matched with at least one biological term among UniProt sequence description, gene ontology (GO) KEGG pathway, or COG information. Among annotated, 4099 (4.1 %) sequences were completely obtained the biological term from all databases and the sequences annotated from individual database were 38,849 (39.6 %) sequences with GO terms, 16,532 (16.8 %) with COG, 7401 (7.1 %) with KEGG pathway respectively.

**Table 2:** Summary of the annotations of the *V. fauriei* transcriptome; GO, Gene Onthology; COG, Cluster Orthologous Groups; KEGG pathway, Kyoto Encyclopedia of Genes and Genomes

Descriptions	Database Ref IDs	No. Sequences
Total Unigenes	-	97,959 (100 %)
Annotated with Uniprot BLAST hits	33,245	47,760 (48.7 %)
Annotated with GO	4908	38,849 (39.6 %)
Annotated with COG	1479	16,532 (16.8 %)
Annotated with KEGG	127	7041 (7.1 %)
Un- annotated	-	50,199 (51.3 %)

**Fig. 3:**
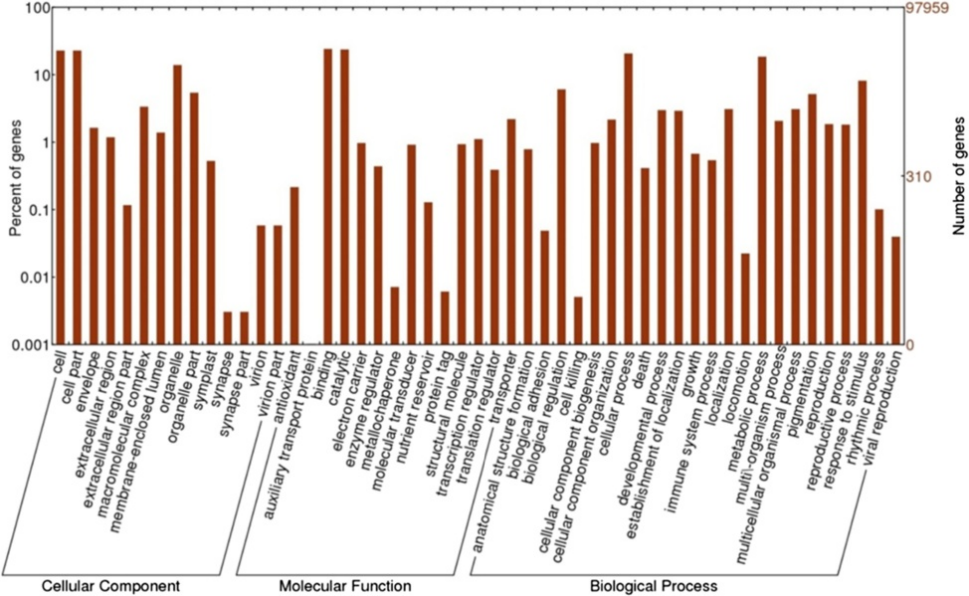
Transcripts grouped to GO subcategories; biological process (BP), molecular functions (MF) and cellular components (CC) with WEGO software. 38,849 unigenes (39.6 % of total) were annotated and the significant GO were plotted in level 1 and 2

**Fig. 4:**
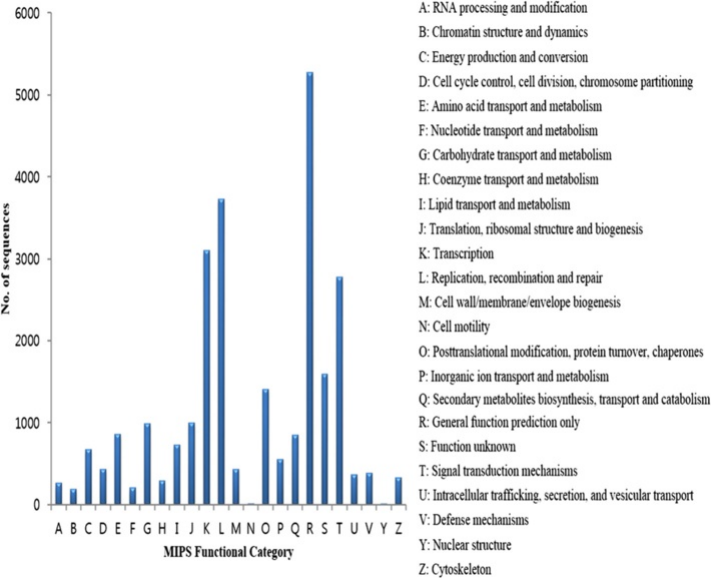
Classification of the clusters of orthologous group (COG) with MIPS functional categories. 16,532 unigenes (16.8 % of total) were annotated and divided into 24 subcategories

Among them, 18,234 (18.6 %) sequences were obtained with the e-value in the range of 0 to 1e-60, and 16,426 (16.8 %) sequences were with ≥70 % similarity (Fig. 2b and c). Also, more than a half of mapped *V. fauriei* transcripts shared annotation information from the three major plant species, i.e. *Vitis vinifera*, *Solanum tuberosum*, and *Populustricho carpa* (Fig. 2d). GO annotations were classified into three subcategories i.e. biological process (BP), molecular functions (MF), and cellular component (CC) (Fig. 3). In the cellular component cluster, cells, cell parts, and organelles were shown to be the top 3 clusters. Among the cluster of molecular functions, binding and catalytic activities were dominant. In the biological process groups, cellular process, metabolic process, and response to stimulus were the largest subcategories. To further characterize the *V. fauriei* transcriptome, sequences were grouped based on MIPS functional categories (Fig. 4). The data comparison enabled the classification of 24 molecular families; the top category was “General function prediction only”. Also, the result shows that only 3 % of sequences belong to secondary metabolite biosynthesis, transport, and catabolism.

Transcriptome is the full set of transcripts including mRNAs, non-coding RNAs, and small RNAs and their quantity for a specific developmental stage or physiological conditions. Transcriptome profiling is widely performed for clarifying all species of transcripts, confirming the transcriptional structure of genes, and quantifying the changing expression levels of each transcript during development and under different conditions [31]. The development of next-generation sequencing (NGS) technology for whole transcriptome sequencing has offered high-throughput, advances in accuracy and sensitivity and decreased costs compared to traditional EST sequencing by Sanger technology [32]. The improvements in genome sequencing technology have provided a valuable opportunity to sequence increasingly large and complex genome [33]. Large scale sequencing of several non-model plants which are potential to investigate the basis of medicinal properties has been already assessed to several species i.e., *Acacia auriculiformis*, *Acacia mangium* [34], *Cajanus cajan* L. [35], *Euphorbia fischeriana* [36], *Myricarubra* [37], and so on. Although this technology has been extensively practiced to various research areas, genomic information of *V. fauriei* is still unknown. Therefore, transcriptome profiling using NGS from non-model plants is useful to generate a reference genome and to provide the basis of finding genes associated with particular important functions [38].

### Digital gene expression and Secondary metabolite related gene

**Fig. 5:**
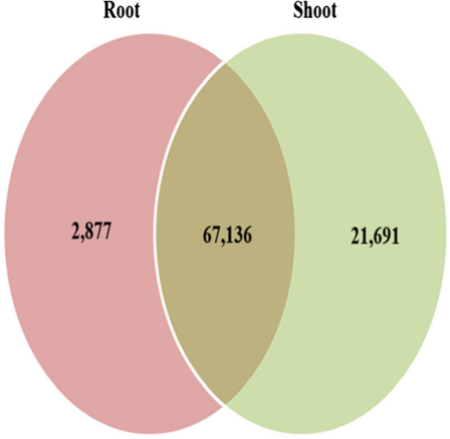
Digital gene expression transcripts with RPKM ≥ 0.3 from each sample, combinations and specific

To analyze the expression profiling of *V. fauriei* transcriptome from root and shoot, all the transcripts were subjected to digital gene expression (DGE) study. First, RPKM values were calculated for each transcript from individual sample. The transcripts with RPKM ≥ 0.3 were considered as being expressed based on previous published information [39]. Root and shoot specific expressions for all combinations within group were plotted in Venn diagram (Fig. 5). Totally, 91,704 (93.6 %) unique transcripts were expressed at least single condition. For individual samples, 70,013 (71.5 %) transcripts and 88,827 (90.7 %) transcripts were expressed in root and shoot, respectively. Among them, 67,136 (68.5 %) transcripts were commonly shown in both root and shoot of *V. fauriei*. Further, expressed transcripts were grouped into more than 2-fold up- or down-regulations (Table 3).

**Table 3:** Changes in gene expression profile between shoot and root libraries

Samples	2 fold changes		
	Up	Down	Total
Root VS Shoot	39,145	6750	45,895

Between the shoot and root libraries, a total of 45,895 DGEs were detected with 39,145 up-regulated genes (higher expression in root) and 6750 down-regulated genes. To better understand the biological functions of the both organ specific transcripts, DGEs were grouped according to KEGG secondary metabolite biosynthesis pathway (Table 4). Results shows that the terpenoids backbone pathway genes and phenylpropanoid related genes were highly upregulated.

**Table 4:** Summary for secondary metabolite genes involved in each biosynthetic pathway

Secondary metabolites	No. unique transcripts	2 Fold
Brassinosteroid biosynthesis	13	10
Flavonoid biosynthesis	11	7
Flavone and flavonol biosynthesis	9	6
Diterpenoid biosynthesis	25	19
Caffeine metabolism	126	59
Isoquinoline alkaloid biosynthesis	158	89
Monoterpenoid biosynthesis	3	3
Carotenoid biosynthesis	46	33
Terpenoid backbone biosynthesis	852	513
Anthocyanin biosynthesis	2	2
Sesquiterpenoid and triterpenoid biosynthesis	16	13
Steroid biosynthesis	140	86
Phenylpropanoid biosynthesis	310	194
Total	1711	1044

Currently, several tools have developed to analyze the gene expression. Especially, digital gene expression (DGE) is getting popular due to its enrichment for a pathway or ontology term by using overlap statistics from variations in the counts of their cognate sequence tags [40, 41]. However, DGE requires a reference sequence to align the relative small read lengths [32]. Indeed, performing DGE and RNA-Seq, which provides reference transcriptome, offers an efficient method to identify the candidate genes encoding enzymes involved in the biosynthesis of secondary metabolites in non-model plants [42].

### Analysis of secondary metabolite biosynthetic genes from *V. fauriei* unigenes

The sequences of secondary metabolite biosynthetic pathway genes were identified in the NGS of the *V. fauriei* database. Several full-length cDNAs encoding *MCT*, *HDS*, *GDS*, *AACT*, *HMGS*, *MK*, *PMK*, *IDI*, and *FDS*, and partial-length cDNAs encoding *DXS*, *DXR*, *CMK*, *MDS*, *HDR*, and *MVD* were isolated from *V. fauriei* in terpenoid biosynthesis (Additional file 4). To confirm this for homology, they were designed as *VfDXS* (489 aa), *VfDXR* (473 aa), *VfMCT* (307 aa), *VfCMK* (310 aa), *VfMDS* (222 aa), *VfHDS* (734 aa), *VfHDR* (446 aa), *VfGDS* (418 aa), *VfAACT* (406 aa), *VfHMGS* (464 aa), *VfHMGR* (582 aa), *VfMK* (389 aa), *VfCMK* (497 aa), *VfMVD* (417 aa), *VfIDI* (235 aa), and *VfFDS* (345 aa) and showed sequence similarities according to BLAST search. Additional file 5 shows the sequences of carotenoid and phenylpropanoid biosynthetic genes identified from NGS data of *V. fauriei*. Among carotenoid biosynthetic genes, full-length cDNAs of *VfPDS* (569 aa), *VfZDS* (579 aa), *VfCHXB* (251 aa), and *VfNCED* (577 aa), and partial-length cDNAs of *VfPSY* (347 aa), *VfCrtISO* (401 aa), *VfLCYB* (363 aa), *VfLCYE* (249 aa), *VfCHXE* (403 aa), *VfZEP* (649 aa), and *VfCCD* (107 aa) were also exhibited. Additionally, full-length cDNAs of *VfCOMT* (240 aa), *VfCHS* (410 aa), *VfF3H* (353 aa), *VfF3’H* (326 aa), and *VfFLS* (332 aa) and partial-length cDNAs of *VfPAL* (468 aa), *VfC4H* (407 aa), *Vf4CL* (406 aa), *VfC3H* (373 aa), *VfHQT* (191 aa), *VfCHI* (172 aa), *VfF3’5’H* (172 aa), *VfFNS* (143 aa), *VfFNS2* (333 aa), *VfGT* (175 aa), and *VfRT* (99 aa) for phenylpropanoid biosynthesis were purified from *V. fauriei*. A BLAST search at the amino acid level showed that secondary metabolite biosynthetic genes from *V. fauriei* exhibited high identity to other orthologous genes.

### Analysis of terpenoid transcript levels and terpenoid content

**Fig. 6:**
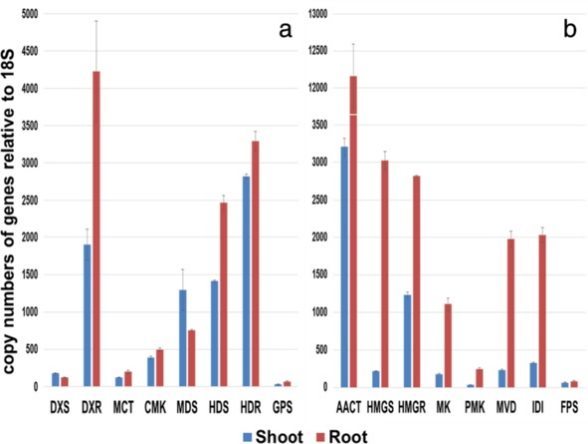
Transcript levels of genes related to terpenoid biosynthetic pathway in shoot and root of *V. fauriei*. Expression levels from three individual replicates were analyzed relative to that of *18S*. Error bars showed SD values. **a** MEP biosynthetic genes, **b** MVA biosynthetic genes

Quantitative real-time PCR analysis was performed to determine the expression levels of terpenoid biosynthetic genes in shoot and root of *V. fauiriei* (Fig. 6). Most of genes which are related to MEP pathway were expressed at the higher level in root than in shoot of *V. fauriei*. However, expression levels of *VfDXR* and *VfMDS* were about 2.23-, 1.17-fold higher in shoot than in root of *V. fauriei* respectively. In contrast, all MVA biosynthetic genes showed a minimum of 1.29- to a maximum of 13.88-fold higher levels in root than in shoot of *V. fauriei*. About 130 volatile compounds were detected in *V. fauriei* by GC and GC/MS analysis. All the reported volatile compounds including terpenes are presented in the Additional files 6 and 7. Among the total volatile compounds, there have been 54 terpenes; 17 monoterpenes, 34 sesquiterpenes, and 3 other terpenes isolated from *V. fauriei*. The results showed that bornyl acetate (13, 24.828 %), cedrol (37, 5.687 %), and α-acrorenol (41, 5.185 %) are the major constituents in root whereas p-cymene (5, 10.879 %) and pentanoic acid (52, 5.564 %) are principal constituents in shoot of *V. fauriei*. Isovaleric acid (53), causing an unpleasant odor, was detected only in the root. HPLC analysis was also done to detect three types of valerenic acid i.e., valerenic acid, acetoxy-valerenic acid, and hydroxyvalerenic acid in both shoot and root of *V. fauriei* (Table 5). The accumulation of valerenic acid and acetoxy-valerenic acid were detected only in root of *V. fauriei*, exhibiting the concentrations of 219.09 μg/g dry weight, 32.22 μg/g dry weights, respectively. However, hydroxyvalerenic acid was not present in the analysis of both shoot and root of this plant.

**Table 5:** Valerenic acid-related compounds content (μg/g dry weight) in root and shoot of *V. fauriei*. Each value is from three determinations ± SD. n.d, not detected

	Valerenic acid	Acetoxy-valerenic acid	Hydroxyvalerenic acid
Root	219.03 ± 6.70	32.22 ± 0.05	n.d
Shoot	n.d	n.d	n.d

Until now, most of the studies have focused on root part of *Valeriana* species, whereas aerial part has been studied very rarely. Leaf contained valeric acid-related compounds as the major compounds [43]. In addition, patchouli alcohol, α-pinene, and β-pinene were shown mainly in the oil of aerial parts of *V. dioschoridis*. Patchouli alcohol and isovaleric acid contributed major level of oil in the aerial parts of *V. celtica* [44, 45]. According to our study, p-cymene and pentanoic acid were detected as major compounds, whereas bornyl isovalerate is present as a little amount in shoot of *V. fauriei*. The major active compounds present in *Valeriana* species such as valerenic acid and its derivatives, and oxygenated sesquiterpenoids were accumulated in roots and rhizomes principally [46]. As expected, valerenic acid-related compounds were shown only in root of *V. fauriei*. Besides, the main compounds of root of this plant were bornyl acetate, cedrol, α-acrorenol. These findings are quite similar to the result of Chen H et al.[47]. They showed that bornyl acetate were the major constituent of the essential oil from the root and rhizomes of *V. alternifolia*.

### Analysis of carotenoid transcript levels and carotenoid content

**Fig. 7:**
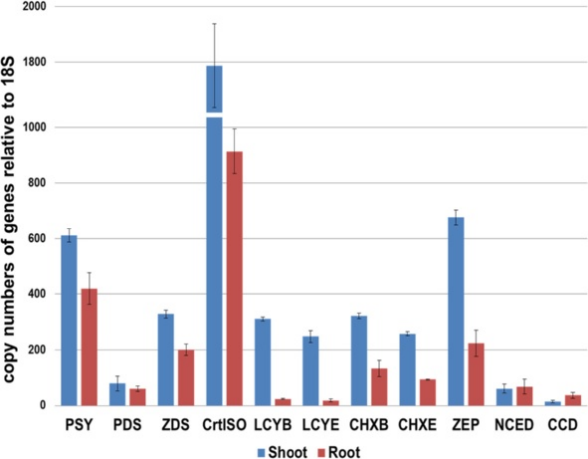
Transcrip levels of genes related to carotenoid biosynthetic pathway in shoot and root of *V. fauriei*. Expression levels from three individual replicates were analyzed relative to that of *18S*. Error bars showed SD values

We conducted real-time PCR to investigate carotenogenic transcript levels between shoot and root of *V. fauriei* and analyzed the carotenoid content by HPLC. Results revealed that the transcripts levels of carotenoid biosynthetic genes were highly expressed as maximum as 13.8-fold at *LCYE* to a minimum of 1.31-fold at *PDS* showing more in the shoot than in the root of *V. fauriei* (Fig. 7). However, expression levels of *NCED* and *CCD*, those are involved in synthesis of aprocarotenoids such as abscisic acid showed a higher level of about 1.11-, and 2.66-fold in root than in shoot respectively. Five different carotenoids i.e., violaxanthin, zeaxanthin, α-carotene, β-carotene, and 9-cis-β-carotene were detectedin *V. fauriei* (Table 6). Higher levels of all carotenoid content were detected in the shoot, whereas only a few quantities of β-carotene and 9-cis-β-carotene were detected in the root of *V. fauriei*.

**Table 6:** Carotenoid content (μg/g dry weight) in root and shoot of *V. fauriei*. Each value is from three determinations ± SD. n.d, not detected

	Violaxanthin	Zeaxanthin	α-carotene	β-carotene	9-cis-β carotene	Total
Root	n.d	n.d	n.d	0.42 ± 0.05	0.05 ± 0.01	0.47 ± 0.05
Shoot	0.16 ± 0.05	10.38 ± 2.08	0.19 ± 0.04	2.99 ± 0.77	0.32 ± 0.11	14.04 ± 3.05

Carotenoids were produced in the photosynthetic organ (shoot) of *V. fauriei* in abundance. In contrast, the production of carotenoid in the underground organ (root) is rarely occurred. The results of the present study correspond well with those found in the earlier experimental studies in Chinese cabbage [48] and bitter melon [49].

### Analysis of phenylpropanoid transcript levels and phenolic compound content

**Fig. 8:**
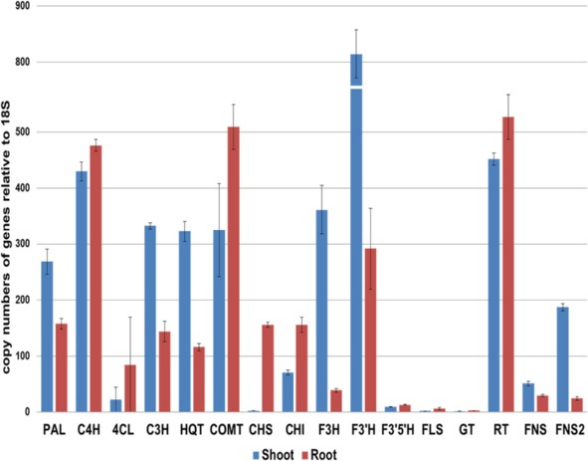
Transcript levels of genes related to phenylpropanoid biosynthetic pathway in shoot of root of *V. fauriei*. Expression levels from three individual replicates were analyzed relative to that of *18S*. Error bars showed SD values

The genes related to phenylpropanoid biosynthetic pathway were examined both in shoot and root of *V. fauriei* (Fig. 8). Six types of phenolic compound such as chlorogenic acid, caffeic acid, ferulic acid, rutin, trans-cinnamic acid, and quercetin were found in *V. fauriei* through HPLC analysis (Table 7). Expression levels of *PAL*, *C3H*, *HQT*, *F3H*, *F3’H*, *FNS*, and *FNS2* were much higher in shoot than in root of *V. fauriei*, whereas a few other genes were expressed higher in root. Every phenolic compound except for chlorogenic acid and quercetin was accumulated highly in the root compared to that of in the shoot. Rutin is synthesized from quercetin by attaching one glucose and one rhamnose, catalyzed by *GT* and *RT*. In root of *V. fauriei*, where the level of expression of *GT* and *RT* genes were higher, also the amount of rutin was higher in the root than that in the shoot. In contrast, quercetin content was more in the shoot. It may be caused due to differential gene activities of *GT* and *RT* as well. Key enzymes for chlorogenic acid synthesis; *C3H* and *HQT* were expressed at the higher level in shoot than in root and lead to a large quantity of chlorogenic acid production.

**Table 7:** Phenolic compounds (μg/mg dry weight) in root and shoot of *V. fauriei*. Each value is from three determinations ± SD. n.d, not detected

	Chlorogenic acid	Caffeic acid	Ferulic acid	Rutin	Trans-cinnamic acid	Quercetin
Root	3.819 ± 0.002	0.193 ± 0.001	0.236 ± 0.004	0.836 ± 0.000	0.004 ± 0.001	0.002 ± 0.001
Shoot	14.726 ± 0.128	0.185 ± 0.001	0.015 ± 0.001	0.288 ± 0.006	0.002 ± 0.000	0.016 ± 0.000

In some studies, phenolic compounds including luteolin, apigenin, quercetin, kaempferol, and ferulic acid have been identified from both above ground biomass and root of *Valeriana* family [46]. Also, Andres Navarrete et al. have demonstrated that chlorogenic acid quantified in a range of 0.2 to 2 % from *V. jatamansi, V. procera, V. edulis, V. sitchensis, and V. officinalis* [50]. The well-known phenolic compounds i.e., trans-caffeic acid and rutin were confirmed in *V. jatamansi* [51]. According to the result of Indra D. Bhatt et al., caffeic acid was highly detected in the aerial part of planted source, whereas chlorogenic acid was found in a higher amount in the root part that in aerial part of *V. jatamansi* [52]. These findings are in contrast to our results. Here in this study, it was demonstrated that caffeic acid shows quite similarly in both root and shoot of *V. fauriei*. Besides, chlorogenic acid was more in shoot of *V. fauriei* where C3H and HQT genes expressed highly. The big differences with regard to chemical constituents indicated that these species are based on morphological [50].

### Metabolic profiles between shoot and root of *V. fauriei* using GC-TOFMS analysis

The primary core metabolites such as organic acids, amino acids, and sugars determined using GC-TOFMS revealed clear metabolite differentiation between various biological samples. ChromaTOF software was used to support peak findings prior to quantitative analysis and for automated deconvolution of reference mass spectra. The NIST and the in-house libraries for standard chemicals were utilized for the identification of the compounds, which 42 metabolites were detected in the samples.

**Fig. 9:**
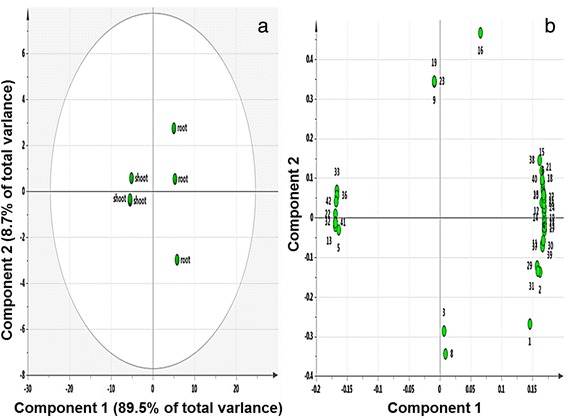
PCA score plots (**a**) and loading plots (**b**) derived from polar metabolites of shoot and root of *V. fauriei*. PC1 and PC2 accounted for >98.2 % of the total variance. The ellipse represents the Hotelling T2 with 95 % confidence in the score plot. The loading plots represent the original variables in the space of the PCs. They reveal the magnitude and direction of correlation of the original variables with the first two PCs. 1, lactic acid; 2, valine; 3, alanine; 4, glycolic acid; 5, serine; 6, ethanolamine; 7, glycerol; 8, isoleucine; 9, proline; 10, nicotinic acid; 11, glycine; 12, succinic acid; 13, glyceric acid; 14, fumaric acid; 15, threonine; 16, β-alanine; 17, malic acid; 18, aspartic acid; 19, methionine; 20, pyroglutamic acid; 21, 4-aminobutyric acid; 22, threonic acid; 23, arginine; 24, glutamic acid; 25, phenylalanine; 26, xylose; 27, asparagine; 28, glutamine; 29, shikimic acid; 30, citric acid; 31, quinic acid; 32, fructose; 33, galactose; 34, glucose; 35, mannose; 36, mannitol; 37, inositol; 38, tryptophan; 39, sucrose; 40, maltose; 41, trehalose; 42, raffinose

Principle component analysis (PCA) widely used to differentiate and correlate the components. Forty two quantified metabolites were normalized to the IS signal intensity were subjected to PCA to outline the differences between the metabolite profiles of the shoot and root samples. As shown in Fig. 9, the first and second principal components of the PCA score plot represented 89.5 and 8.7 % of the total variance of the samples, respectively. The PCA results clearly showed the absence of marked variances between samples of same tissue. The corresponding loading was negative for serine, glyceric acid, threonic acid, fructose, galactose, mannitol, trehalose, and raffinose, indicating that the 8 metabolites were higher in shoot than in root. Interestingly, glucose, phenylalanine, and shikimic acid associated with terpenoid, carotenoid, and phenylpropanoid biosynthetic pathways showed higher levels in root than in shoot. Combined transcriptome and targeted metabolic profiling data possibly provides correlations between genes and metabolites in given biological systems on a whole-genome or metabolome scale [53]. Also, NGS with metabolomics tools can provide insight into the nature of transcript-metabolite networks because of the increased ease and efficiency [54].

In further study, we expect that tissue-specific transcript profiling can provide insights into biological, functional differences between independent transcripts of both shoot and root of *V. fauriei*. In addition, there is an opportunity to produce medicinal substances for industrial purposes in both quality and quantity through genetic engineering.

## Conclusions

In present study, we obtained a total of 97,959 unigenes using Illumina HiSeq system from *V. fauriei*. Among them, there were 47,760 annotated genes with Uniprot BLAST hits and mapped to GO, COG, and KEGG pathway. Individually, 70,013 transcripts and 88,827 transcripts were expressed in root and shoot, respectively of *V. fauriei*. Transcripts were highly involved in terpenoid backbone biosynthesis and phenylpropanoid biosynthesis compared to other biosynthesis by grouping into KEGG secondary metabolite biosynthetic pathway. Therefore, we found that de novo transciptome sequencing with DGE analysis is a modern novel technique for the identification of specific genes for the candidate enzymes involved in the biosynthesis of secondary metabolites in *V. fauriei*. We also investigated the expression of genes suggested an association with secondary metabolism in root and shoot of *V. fauriei*. Most of genes related to terpenoid biosynthesis are highly expressed in root than in shoot of *V. fauriei*. Also, we confirmed the presence of about 130 volatile compounds those are isolated from *V. fauriei* using GC/MS and also it is mentionable that valerenic acid-related compounds are shown only in the root of this plant. However, all carotenoid biosynthetic genes except *NCED* and *CCD* were expressed in a higher rate in the shoot than that of in the root of this plant. In addition, carotenoids were commonly accumulated in shoot. A couple of phenylpropanoid biosynthetic genes are expressed higher in the shoot and others are expressed in a higher rate in the root of *V. fauriei* showing in different amounts.

According to transcriptome databases, the comparison of gene expression and metabolite accumulation were achieved in this study. The transcriptome analysis for *V. fauriei* gives an opportunity to characterize genes leading to the synthesis of secondary metabolites, compounds of interest.

## Methods

### Plant materials and RNA isolation

*V. fauriei* seeds were collected from Rural Development Administration (RDA, Korea) and *V. fauriei* plants were established in a greenhouse at the experimental farm of Chungnam National University (Daejeon, Korea). Any temperature condition or additional illumination has not been regulated to the cultures. The plantlets were exposed to outdoor conditions in the greenhouse for 2 months between 19.05.2014 and 20.07.2014. Average temperature in this season was 21.8 °C. Plant materials were excised from 2-month-old young seedling plants and dissected into shoot (leave and stems) and root (roots and rhizomes). The separated plant parts were washed thoroughly with sterile water and frozen in liquid nitrogen immediately and stored at −80 °C. Shoot and root of *V. fauriei* were ground with liquid nitrogen. Total RNA was extracted from each part of *V. fauriei* separately using the Total RNA Mini Kit (Geneaid, Taiwan) by following the catalogues instruction and the concentrations were determined by agarose gel electrophoresis and NanoVue Plus Spectrophotometer (GE Healthcare Bio-Science Crop, USA) respectively. All samples were harvested and RNA isolation was performed in triplicate.

### Sequence analysis

Illumina sequencing data was used for the identification of genes involved in terpenoid backbone, carotenoid, and phenylpropanoid metabolic biosynthetic pathway. The individual candidate gene name was searched using the functional annotation file. The selected amino acid sequences of the genes were analyzed for homology using BLAST at the NCBI Genbank database (http://www.ncbi.nlm.nih.gov/BLAST).

### Next generation sequencing of transcriptome

To obtain high-throughput transcriptome data of *V. fauriei*, we implemented Illumina-based NGS sequencing. Total RNAs were quantified using Nanodrop spectrophotometer (Thermo Scientific) and quality-assessed by RNA 6000 Nano assay kit (Agilent) and Bioanalyser 2100 (Agilent). NGS sequencing libraries were generated from one microgram of total RNA using Truseq RNA Sample Prep Kit (Illumina) according to the manufacturer’s protocol. In brief, the poly-A containing RNA molecules were purified using poly-T oligo attached magnetic beads. After purification, the total poly A+ RNA was fragmented into small pieces using divalent cations under elevated temperature. The cleaved mRNA fragments were reverse transcribed into first strand cDNA using random primers. QiaQuick PCR extraction kit was used for the purification of the shorts fragments and further resolved with EB buffer for end reparation and addition of poly (A). After that, the short fragments with poly (A) tail were interlinked with sequencing adapters. Each library was separated by adjoining distinct MID tag. The resulting cDNA libraries were then paired-end sequenced (2x101bp) with Illumina HiSeq™ 2500 system.

### De novo assembly

Complete paired end sequences were obtained as individual fastq files (forward and reverse) from the images by CASAVA (version 1.8.2) base calling software with ASCII Q-score offset 33. Adaptor sequences and low quality bases with PHERD scores (Q) ≤ 20, were removed. Repeat sequences in raw reads were masked by using Repeat Makser against *Arabiopdopsis* Rep base database. Simple sequence repeats and low complexity sequences were also masked by using SSRIT and DUST software respectively [55]. Finally, masked sequences were subjected to *de novo* assembly by using CLC Assembly Cell v.4.0 (CLCBio, Inc. Denmark) with customized parameters. To optimize transcriptome assembly, a set of assembly was done with word size of 21 to 63 with increment of 2 (data not shown). From the different assemblies, the best one was selected based on the sequence mapping coverage which was assessed by reference mapping of clean reads to an individual assembly with CLC mapper with length fraction 80 % and similarity 90 %. Based on the reference mapping results, the assembly with word size 63 was chosen as the best assembly. To remove the isoforms and obtain the non-redundant sequence set from the selected assembly contigs, CAP3 [56]was performed with default parameters. Finally, results from CAP3 (singletons and contigs) were merged together to generate reference transcriptome assembly and renamed with unique sequence identifiers.

### Functional annotations

Non-redundant transcripts (*per se* reference transcriptome) were subjected to functional annotations by sequence homology search against biological databases such as GO, KEGG, and COG. Sequences were first mapped to UniProt database using BLASTX with e-value cut-off of 1e-5. Biological descriptions, GO terms, KEGG, and COG identifiers were transferred from the best-matched UniProt entry among the mapped sequence for each sequence. Then, the gene ontology functional classifications such as biological process (BP), molecular function (MF) and cellular components (CC), were grouped according to the GO hierarchy level 2 and the distributions were plotted using WEGO [57]. Similarity, e-value, and species distributions were calculated from BLAST results. Transcripts were also grouped according to KEGG map IDs. Finally, sequence descriptions and references were collected from UniProt database using biopython module.

### Digital gene expression (DGE) profiling and selection of transcripts involved in secondary metabolic pathway

To characterize the quantitative expression profile of individual sequence, the clean sequence reads from two libraries (root and shoot) were mapped individually to the reference transcriptome using CLC mapper with 90 % similarity and 80 % length fraction respectively. Based on the read count to each transcript, the reads per kilo base per million (RPKM) value was calculated. RPKM = (10^9^ * C) / N * L. C is number of mapped reads per sequence, N is total number of mapped reads and L is length of the sequence [58]. The statistical significance difference between the expression levels of each transcript within each pair of conditions were assessed by Audic and Claveriae’ method and false discovery (FDR) rate control [40, 59]. RPKM values were taken to Gene Spring 12.5 GX to calculate the fold changes (FC) through 2 libraries with default parameter. Fold changes were calculated for category such as root vs Shoot. Finally, sequence were filtered with RPKM ≥0.3, FDR >0.001 and FC ≥2.0 [42]. Annotations obtained from the UniProt database such as gene descriptions, GO terms and sequence descriptions were manually examined from the known keywords (referred by published articles and KEGG pathways) for the candidate gene selections of secondary metabolism related.

### cDNA synthesis and quantitative real-time PCR

cDNA was synthesized from 1 μg of total RNA using the ReverTra Ace-α Kit (Toyobo, Osaka, Japan). For all target genes and the *18S* gene, an internal reference, primers were designed with GenScript Real-time PCR (TaqMan) Primer Design (www.genscipt.com) to conduct quantitative real-time PCR (Additional file 8). Quantitative real-time PCR was performed in a BIO-RAD CFX96 Real-time PCR system (Bio-Rad Laboratories, Hercules, CA) with the 2X Real-Time PCR smart mix (Solgent Co., Ltd. Daejeon, Korea) under the following conditions: pre-denaturation at 95 °C for 15 min, denaturation for 20 s at 95 °C, and reaction cycle was repeated for 39 cycles at annealing for 40 s, 20 s at 72 °C and final extension at 72 °C for 10 min. The differences between treatment means were evaluated from tree independent replicates for each sample. Then, average mean value and standard deviation value from three replicates of respective samples were analyzed.

### Analysis of GC and GC-mass spectrometry

Ten gram of the fresh shoot and root of *V. fauriei* were weighed and transferred into 25 ml headspace vials. A fused-silica fiber covered with a 75 μm layer of carboxen/polydimethylsiloxane (CAR/PDMS) was used for absorption of the volatile compounds in the fresh shoot and root. The vials containing the samples and the solvents were kept at 25 °C for 20 min and was then removed from the vial and injected into the GC where analysis was performed at 250 °C for 3 min.

GC analysis was performed using an Agilent 6890 N GC mainframe equipped with an HP-5 (30 m × 0.32mmID, film thickness 0.25 μm) fused-silica capillary column (Agilent, USA) and a flame ionization detector. The injector and detector temperatures for each analysis were 250 and 280 °C, respectively. The carrier gas was nitrogen at a flow rate of 1.0 mL · min^-1.^ The column temperature was maintained at 50 °C for 5 min and afterward programmed as follow : increase from 50 to 260 °C at a rate of 3 °C · min^−1^, increase from 260 to 280 °C at a rate of 10 °C · min^−1^ and hold at 280 °C for 5 min.

GC-MS analysis was performed on a GC/MSD Polaris Q (thermoFinnigan, USA) with an HP-5 (30 m × 0.32 mm ID, film thickness 0.25 μm) fused-silica capillary column (Agilent, USA). Helium was used as the carrier gas at a flow rate of 1.0 mL · min^−1^. For GC-MS detection, an electron ionization system with system energy 70 eV, trap current 250 μA, and ion source temperature 200 °C was used. The oven temperature program was the same as that described for GC, and injections were used in the split less mode. To identify of samples, components compared of mass spectra with the NIST and WILLY library data of the GC-MS system and with data from the literature. Total ion current chromatograms were recorded in a mass rage of 40–400 amu.

### High performance liquid chromatography analysis

Collected samples were dried in the freeze-dryer at −80 °C for 3 days. Dried samples were ground into a fine powder using a mortar and pestle.

#### Quantification of valerenic acid and its derivatives

One gram of powdered samples was extracted with 10 mL of 90 % (v/v) methanol at room temperature for 30 min and the extracts were centrifuged at 12,000 rpm for 10 min. This step was repeated for three times. Thereafter, the final extract were evaporated using LABOROTA 4000 (Heidolph, Germany) and filtered with a 0.45-μm Acrodisc syringe filter (Pall Corp.; Port Washington, NY), for HPLC analysis. HPLC analysis was performed with a C18 column (μBondapak™ C18 10 μm 125Å 3.9 × 300 nm column). The mobile phase was a gradient prepared from mixtures of acetonitrile and 0.25 % phosphoric acid and the column temperature was maintained at 30 °C. The flow rate was maintained at 0.7 mL/min. Injection volume of 20 μL and 221 nm wavelengths were used for detection. The compounds of standard were determined by using a standard curve.

#### Carotenoid

For carotenoids quantification, 300 mg of *V. fauriei* samples were mixed with 3 ml of ethanol containing 0.1 % ascorbic acid (w/v). This mixture was mixed throughly for a while, then incubated in a water bate at 85 °C for 5 min. In subsequent step, separately 120 μl of potassium hydroxide (80 % w/v) was added to saponify any potential interfering oils. After vortex, incubated at 85 °C for 10 min again and immediately keep it ice for 5 min. Separately, 1.5 ml of cold deionized water was mixed with 0.05 ml of b-Apo-80-carotenal (1.25 μg) was added to mixture as an internal standard. 1.5 ml of hexane was used for the extraction of extraction of complete carotenoids. The isolated carotenoids layers were separated by centrifugation at 1200 rpm for 10 min. The resulting extracts were freeze-dried by passing nitrogen gas and resuspended in 50:50 (v/v) dichloromethane/methanol. The carotenoids were separated on an Agilent 1260 HPLC system. The separating solvent consists of mixture of solvent A (methanol/water (92 % v/v) including 10 mM ammonium acetate) and solvent B consisted of 100 % methyl tert-butyl ether (MTBE). The separating solvents were flow at 1.0 ml/min and the injection sling was 20 μl. Samples were eluted with the following gradient: 0 min, 90 % A/10 % B; 20 min, 83 % A/17 % B; 29 min, 75 % A/25 % B, 35 min, 30 % A/70 % B; 40 min, 30 % A/ 70 % B; 42 min, 25 % A/75 % B; 45 min, 90 % A/10 % B; 55 min, 90 % A/10 % B. With the help of previous guidelines, based on the retention time and UV-visible spectrum data, carotenoids were identified.

#### Phenolic compound

Phenolic compounds extracted by mixing 100 mg samples with 80 % methanol at room temperature for 1 h in a 10 ml sterile test tube. After proper mixing, the crude mixture was centrifuged and the supernatant was filtered through 0.45 μm filter before HPLC analysis. Individual phenolic compounds were eluted, separated and quantified in a Futecs model NS-4000 HPLC apparatus (Daejeon, Korea). The HPLC analysis was performed in C18 column (maintained at 30 °C) and the phenolic compounds were detected at 280 nm. Gradient mobile phase system [water: acetic acid (98:2 v/v)] was used for the elution of the phenolic compounds. The gradient solvents were flown at 1.0 ml/min and 20 μl of the sample was used for injection. The contents of individual phenol compounds were calculated using a standard calibration curve. All samples were run in triplicate.

### GC-TOFMS analysis of polar metabolites

The extraction of polar metabolite was performed as described by Kim et al. (2013) [60]. The sample preparation methods, chemicals and reagents, esterification procedures, GC-TOFMS instrument operating conditions, analytical methods for the separation of the samples and the scanning and the detection of the compounds rang were as implemented as described in our previous research paper [58].

### Statistical analysis

In *V. fauriei* samples, 42 metabolites were identified by GC-TOFMS. The quantitative calculations of all analytes were based on the peak area ratios relative to that of the IS. The relative quantification data acquired from GC-TOFMS were subjected to PCA (SIMCA-P version 13.0; Umetrics, Umeå, Sweden) to evaluate the relationships in terms of similarity or dissimilarity among groups of multivariate data. The PCA output consisted of score plots to visualize the contrast between different samples and loading plots to explain the cluster separation. The data file was scaled with unit variance scaling before all the variables were subjected to the PCA.

### Ethics approval and consent to participate

Not applicable.

### Consent for publication

Not applicable.

### Availability of data and material

The datasets supporting the conclusions of this article are included within the article and its additional files.

## Additional files

Additional file 1: Terpenoid biosynthesis pathway in plants. *AACT*, acetoacetyl-CoA thiolase; *CMK*, 4-(cytidine 5’-diphospho)-2-C-methyl-D-erythritol kinase; DMAPP, dimethylallyl diphosphate; *DXR*, 1-deoxy-D-xylulose 5-phosphate reductoisomerase; *DXS*, 1-deoxy-D-xylulose 5-phosphate synthase; *FPS*, farnesyl diphosphate synthase; *GPS*, geranyl diphosphate synthase; *HDR*, 4-hydroxy-3-methylbut-2-enyl diphosphate reductase; *HDS*, 4-hydroxy-3-methylbut-2-enyl diphosphate synthase; *HMGR*, hydroxymethylglutaryl-CoA reductase; *HMGS*, hydroxymethylglutaryl-CoA synthase; *IDI*, isopentenyl diphosphate isomerase; IPP, isopentenyl diphosphate; *MCT*, 2-C-methyl-D-erythritol 4-phosphate cytidylyltransferase; *MDS*, 2-C-methyl-D-erythritol 2,4-cyclodiphosphate synthase; *MVD*, mevalonate diphosphate decarboxylase; *MK*, mevalonate kinase; *PMK*, 5-phosphomevalonate kinase. (PDF 160 kb) Additional file 2: Carotenoid biosynthesis pathway in plants. *CCD*, carotenoid cleavage dioxygenases; *CHXB*, β-ring hydroxylase; *CHXE*, ε-ring hydroxylase, *CrtISO*; carotenoid isomerase; *LCYB*, lycopene β-cyclase; *LCYE*, lycopene ε-cyclase; *NCED*, nine-cis-epoxycarotenoiddioxygenanses; *PDS*, phytoene desaturase; *PSY*, phytoene synthase; *ZDS*, ζ-carotene desaturase; *ZEP*, zeaxanthin epoxidase. (PDF 154 kb) Additional file 3: Phenylpropanoid biosynthesis pathway in plants. *4CL*, 4-coumaroyl CoA ligase; *C3H*, p-coumarate-3-hydroxylase; *C4H*, cinnamate 4-hydroxylase; *CHI*, chlacone isomerase; *CHS*, chalcone synthase; *COMT*, caffeate O-methyltransferase; *F3H*, flavone-3-hydroxylase; *F3’H*, flavonoid-3’-hydroxylase; *F3’5’H*, flavonoid 3’5’-hydroxylase, *FLS*, flavonol synthase; *FNS I, II*, flavone synthase I, II; *GT*, flavonoid 3-O-glucosyltransferase; *HQT*, hydroxycinnamoyl-CoA quinate hydroxyl cinnamoyl transferase; *PAL*, phenylalanine ammonia-lyase; *RT*, flavonol 3-O-glucoside L-rhamnosyltransferase. (PDF 147 kb) Additional file 4: Comparison of terpenoid biosynthetic genes of *V. fauriei* with the most orthologous genes. (XLS 34 kb) Additional file 5: Comparison of carotenoid and phenylpropanoid biosynthetic genes of *V. fauriei* with the most orthologous genes. (XLS 40 kb) Additional file 6: Amount of terpenes in *V. fauriei* by solid-phase micro-extraction (%) (XLS 31 kb) Additional file 7: Amount of other volatile compounds in *V. fauriei* by solid-phase micro-extraction (%). (XLS 34 kb) Additional file 8: Primers used to amplify *V. fauriei* genes. (XLS 40 kb)
