# Supplementary figures and images for: Transcriptome and metabolome analysis in shoot and root of Valeriana fauriei
Source: BMC Genomics. 2016 Apr 23;17:303. doi: 10.1186/s12864-016-2616-3 (PMC4842265; doi:10.1186/s12864-016-2616-3)

**MVA pathway**  
(Cytosol)

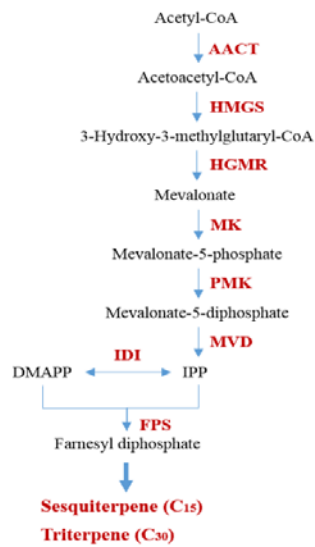

**MEP pathway**  
(Plastid)

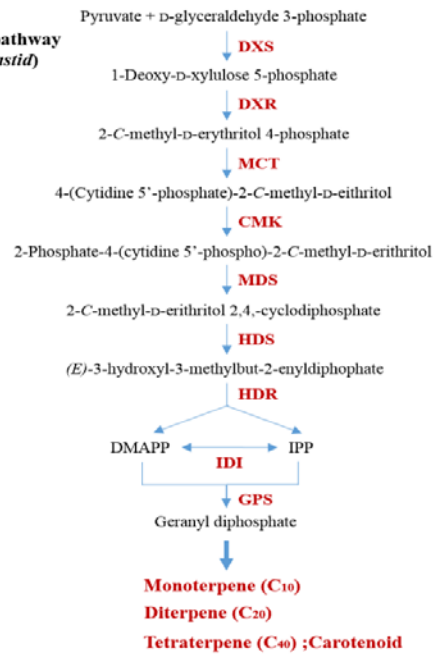

Supplement: Additional file 1: — Terpenoid biosynthesis pathway in plants. AACT, acetoacetyl-CoA thiolase; CMK, 4-(cytidine 5’-diphospho)-2-C-methyl-D-erythritol kinase; DMAPP, dimethylallyl diphosphate; DXR, 1-deoxy-D-xylulose 5-phosphate reductoisomerase; DXS, 1-deoxy-D-xylulose 5-phosphate synthase; FPS, farnesyl diphosphate synthase; GPS, geranyl diphosphate synthase; HDR, 4-hydroxy-3-methylbut-2-enyl diphosphate reductase; HDS, 4-hydroxy-3-methylbut-2-enyl diphosphate synthase; HMGR, hydroxymethylglutaryl-CoA reductase; HMGS, hydroxymethylglutaryl-CoA synthase; IDI, isopentenyl diphosphate isomerase; IPP, isopentenyl diphosphate; MCT, 2-C-methyl-D-erythritol 4-phosphate cytidylyltransferase; MDS, 2-C-methyl-D-erythritol 2,4-cyclodiphosphate synthase; MVD, mevalonate diphosphate decarboxylase; MK, mevalonate kinase; PMK, 5-phosphomevalonate kinase. (PDF 160 kb) [file 12864_2016_2616_MOESM1_ESM.pdf]

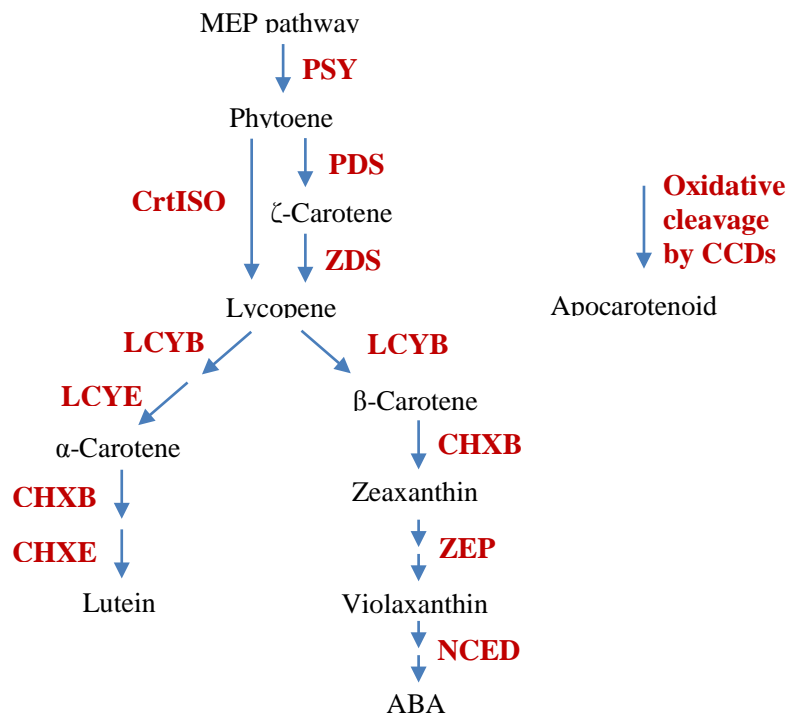

Supplement: Additional file 2: — Carotenoid biosynthesis pathway in plants. CCD, carotenoid cleavage dioxygenases; CHXB, β-ring hydroxylase; CHXE, ε-ring hydroxylase, CrtISO; carotenoid isomerase; LCYB, lycopene β-cyclase; LCYE, lycopene ε-cyclase; NCED, nine-cis-epoxycarotenoiddioxygenanses; PDS, phytoene desaturase; PSY, phytoene synthase; ZDS, ζ-carotene desaturase; ZEP, zeaxanthin epoxidase. (PDF 154 kb) [file 12864_2016_2616_MOESM2_ESM.pdf]

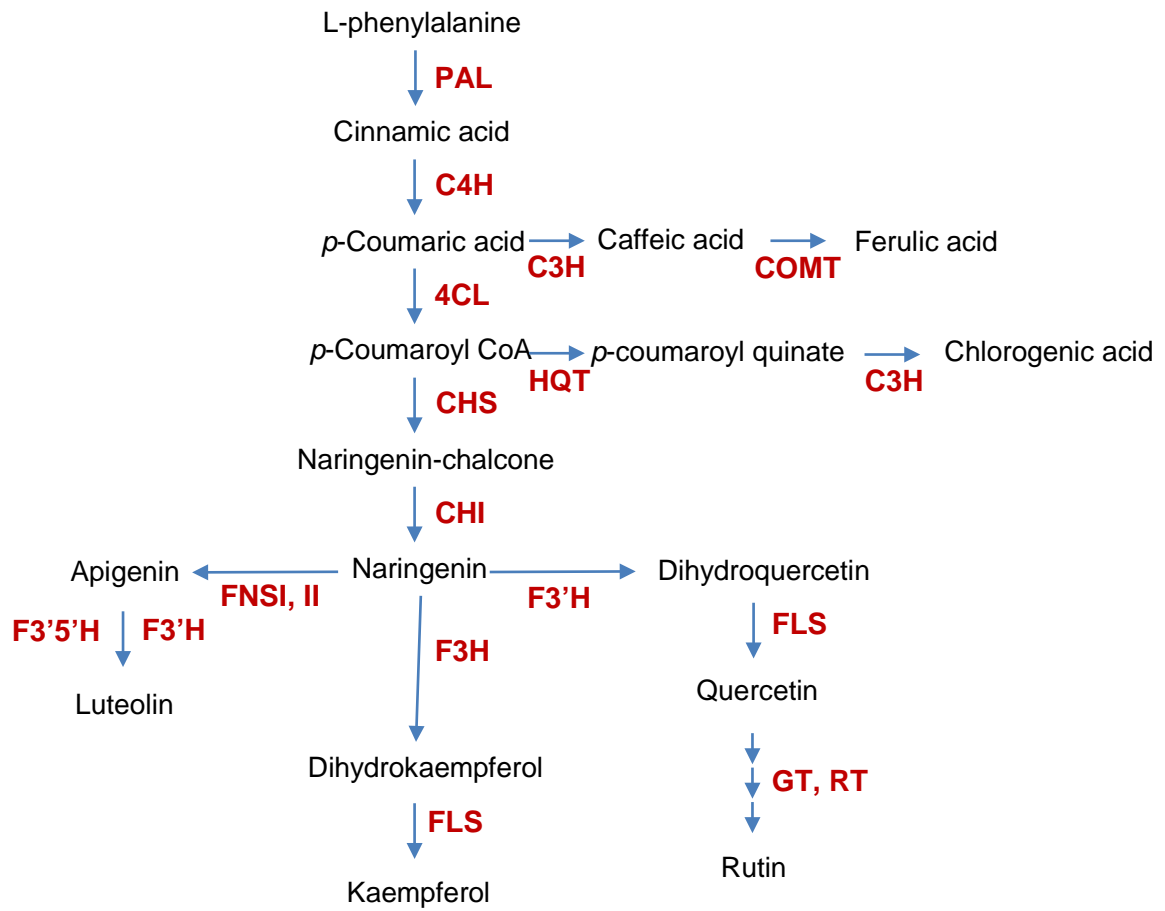

Supplement: Additional file 3: — Phenylpropanoid biosynthesis pathway in plants. 4CL, 4-coumaroyl CoA ligase; C3H, p-coumarate-3-hydroxylase; C4H, cinnamate 4-hydroxylase; CHI, chlacone isomerase; CHS, chalcone synthase; COMT, caffeate O-methyltransferase; F3H, flavone-3-hydroxylase; F3’H, flavonoid-3’-hydroxylase; F3’5’H, flavonoid 3’5’-hydroxylase, FLS, flavonol synthase; FNS I, II, flavone synthase I, II; GT, flavonoid 3-O-glucosyltransferase; HQT, hydroxycinnamoyl-CoA quinate hydroxyl cinnamoyl transferase; PAL, phenylalanine ammonia-lyase; RT, flavonol 3-O-glucoside L-rhamnosyltransferase. (PDF 147 kb) [file 12864_2016_2616_MOESM3_ESM.pdf]
